# Food insecurity and its severity among adults receiving antiretroviral therapy in health facilities, northcentral Ethiopia: a multi-facility-based cross-sectional study

**DOI:** 10.3389/fpubh.2024.1380958

**Published:** 2024-07-22

**Authors:** Dube Jara Boneya, Ahmed Ali Ahmed, Alemayehu Worku Yalew

**Affiliations:** School of Public Health, College of Health Sciences, Addis Ababa University, Addis Ababa, Ethiopia

**Keywords:** Food insecurity, HIV/AIDS, clinical progression, antiretroviral therapy, adults

## Abstract

**Background:**

Food insecurity plays a crucial role in predicting the spread of HIV due to the adverse effects of coping mechanisms adopted to mitigate it. However, there is a scarcity of context-specific evidence regarding food insecurity among HIV-infected adults. Therefore, this study aimed to assess the context-specific magnitude of food insecurity and associated factors among adults receiving antiretroviral therapy (ART) in health facilities in the North Shewa Zone, Ethiopia, ultimately contributing to the achievement of the 95–95-95 HIV treatment target in the local context.

**Methods:**

A multi-facility cross-sectional study was conducted among 865 HIV-infected adults receiving ART and being followed up for their treatment. We included health facilities that provide ART, including four hospitals and six health centers. A log-binomial regression model was fitted to identify the association between food insecurity and independent variables. Adjusted prevalence ratios (APRs) with a 95% confidence interval were computed to measure the strength of the association.

**Results:**

In this study, 290 (33.7, 95% CI: 30.60, 36.91) of the HIV-infected adults studied had food insecurity during their treatment and follow-up, of which 152 (52.41, 95% CI: 46.64, 58.13) and 110 (37.93%, CI: 32.50, 43.68) of them were found to have severe and moderate forms of food insecurity, respectively. We found that being younger (APR = 2.27, 95% CI: 1.12, 4.60), being female (APR = 1.87, 95% CI: 1.03, 3.39), lacking formal education (APR = 10.79, 95% CI: 14.74, 24.58), having lower educational status (APR = 5.99, 95% CI: 2.65, 13.54), being a daily laborer (APR = 6.90, 95% CI: 2.28, 20.85), having low monthly income (APR = 1.89, 95% CI: 1.11, 3.22), advanced WHO clinical stage (APR = 2.34, 95% CI: 1.08, 5.10), and receiving ART for less than 4 years (AOR = 2.28, 95% CI: 1.09, 4.74) were significantly associated with a high proportion of food insecurity among HIV-infected adults.

**Conclusion:**

The magnitude of food insecurity among HIV-infected adults receiving ART was high, with an extremely high magnitude of severe food insecurity. The finding suggests the need for culture- and context-specific nutritional interventions to address the gender dynamics of food insecurity, attention to the early stage of ART, and the integration of strategies to improve educational status and enhance income-generation activities of HIV-infected adults. This requires an emphasis on the link between food insecurity and HIV in Ethiopia’s national food and nutrition policy.

## Introduction

Globally, human immunodeficiency virus (HIV) and acquired immunodeficiency syndrome (AIDS) continue to pose critical health problems at an alarming rate, particularly in developing countries (1). By the end of 2022, there were 1.3 million new HIV infections worldwide, with 39 million people living with HIV (PLHIV), of which 37.5 million HIV infections were among adults (2). Additionally, there were 500, 000 new HIV infections in Eastern and Southern Africa, with a total of 20.8 million PLHIV (3, 4).

In Ethiopia, there were 7,194 new HIV infections, for a total of 603,537 PLHIV in 2023 (5, 6). The estimated HIV prevalence (aged 15–49) was 0.91%, and the estimated AIDS deaths were 9,984 in 2023 (7, 8). This requires a holistic and comprehensive approach in addition to the gains achieved through antiretroviral therapy (ART) in areas where HIV prevalence is high, such as sub-Saharan Africa, including Ethiopia (9).

As one of the strategic approaches, UNAIDS developed a new set of ambitious targets that call for action to reach 95–95-95: 95% of all PLHIV to know their HIV status, 95% of all people with diagnosed HIV infection to receive sustained ART, and 95% of all people receiving ART to have viral suppression by 2025. This aims to close the testing gap and protect the health of millions of PLHIV who are still not accessing treatment, including in Ethiopia (10, 11).

Ethiopia has made excellent progress toward achieving the 90–90-90 treatment goal of the 2016–2021 strategy, particularly the second and third 90s among adults, in which 79% of estimated PLHIV who know their status were on ART, 90% were on ART and 91% were virally suppressed, with marked regional variations in ART coverage (12). The HIV/AIDS National Strategic Plan (NSP) for Ethiopia 2021–2025 indicates that the country is committed to achieving the global new and ambitious 95–95-95 HIV prevention roadmap, with a particular focus on reaching 95% coverage of ART and viral suppression nationally, across all age groups (13).

Food insecurity, defined as “the limited or uncertain availability of nutritionally adequate, safe foods or the inability to acquire personally acceptable foods in socially acceptable ways,” is an important promoter of HIV transmission and disease progression (1) and the leading cause of morbidity and mortality (14). It can have an impact on addressing the 95–95-95 NSP treatment targets that Ethiopia is committed to achieving, which is critical for treatment programs to establish community-centered strategies and systems. Despite this fact, global evidence indicates that approximately 2.4 billion people worldwide, including PLHIV, lack access to adequate food, with 30% experiencing moderate or severe food insecurity. Furthermore, over 3.1 billion people were unable to afford a nutritious and healthy diet, with 78% of them residing in Africa in 2022. This situation is more exacerbated among PLHIV as a result of various contributing factors, including the infection process itself (15). In this regard, studies have noted that the prevalence of food insecurity is high among PLHIV in both resource-rich settings, where its prevalence ranges from 53.6 to 71% (16–18), and resource-poor settings, such as countries of Africa (19–22), where the prevalence of food insecurity ranges from 49.1 to 84.6%. In Ethiopia (23–32), the prevalence ranges from 35.2 to 92.82%.

Studies have indicated that educational status, gender, occupation, food assistance, delaying and skipping drugs, longer duration of ART, missing clinical appointments, and exchanging sex for food are contributing factors for higher food insecurity among PLHIV (18, 20, 22, 33–35).

In Ethiopia, studies indicated that the economic status, educational status of PLHIV, absence of food support, unemployment, residence, WHO clinical stage, poor adherence to treatment, and inadequate household dietary diversity were found as contributing factors for food insecurity, while these studies suggested further investigation on the direction of effect about sample variation (26, 28–30, 36). PLHIV and receiving ART need a sufficient amount of food to maintain a healthy dietary intake and cope with drug side effects. Food insecurity can pose significant challenges to the proper management of food and nutrition implications of ART (37). Furthermore, the review of the National Food and Nutrition Policy of Ethiopia indicates that due emphasis was not given to the link between food insecurity and HIV (38).

It is apparent that few studies were conducted on food insecurity and its associated factors among HIV-infected adults in low- and middle-income countries, including Ethiopia. Very little evidence has been documented online for researchers and policymakers. Therefore, the main objective of this study was to assess the magnitude of food insecurity and its severity and to identify factors associated with food insecurity, among adults receiving ART in health facilities, in Northcentral Ethiopia.

## Materials and methods

### Study design, setting, and period

A multi-facility cross-sectional study was conducted as part of a multi-center prospective follow-up study in health facilities in North Shewa Zone, Oromia, Ethiopia. The Zone has 16 districts (4 town administrations and 12 rural districts) with an estimated total population of 1,431,305 (717,552 male individuals and 713,753 female individuals) (39). The Zone has 5 hospitals (1 referral hospital and 4 primary hospitals) and 64 health centers. The study was conducted in 10 health facilities (4 hospitals and 6 health centers) that have been providing ART services to HIV-infected people with established ART clinics between January 2021 and April 2022.

### Population and eligibility criteria

Adults infected with HIV and receiving ART who had follow-up for their treatment in North Shewa Public Health Facilities were considered the source population. All PLHIV who were receiving ART and had follow-up for their treatment in selected health facilities were the study population. All PLHIV who were receiving ART and whose ages were greater than 18 years, regardless of their treatment regimen and duration of follow-up, were included in the study. Patients with other concomitant chronic diseases, such as heart disease, hypertension, diabetes mellitus, and others that can suppress the immune system and deteriorate their nutritional status, including pregnant women who started ART, were excluded.

### Sample size determination and sampling procedure

The current study is part of a multi-center prospective follow-up study that aimed to assess the effect of food insecurity on the clinical progression of HIV/AIDS and CD4 count change among adults receiving ART in North Shewa Zone Health Facilities. The required sample size was calculated using two population proportion formulas for the difference between the two populations, considering major exposure variables of food insecurity after reviewing different literature. Therefore, the required sample size was calculated using STATCALC application of Epi-info version 7.0 statistical software (40), considering a confidence level of 95%, power of 80%, an adjusted prevalence ratio (PR) of 2.4 for food support, a one-to-one allocation ratio of unexposed to exposed (1:1), a percentage of the outcome variable in unexposed (food insecure household receiving food support) of 3.4% (28), and 5% non-response rate. The final sample size was determined to be 865 using the two-population proportion formula below (41).

$n=\frac{\left(\frac{z\alpha }{2}\sqrt[{\;2}]{\left(1+\frac{1}{r}\right)p\left(1-p\right)}+Z\beta \sqrt[{\;2}]{(p1\;\left(1-p1\right)+\frac{p2\left(1-p2\right)\;}{r}}2\right)}{\left(p1-p2\right)2}=824$ .

After adding in a 5% non-response rate, the sample size remained at 865.

Hospitals and health centers in North Shewa that provided ART and care were identified. All hospitals and six health centers that provided care were included in the study. The calculated sample was proportionally allocated to each hospital and health center based on the size of the patient population. Finally, the data were collected from the participants selected, using simple random sampling from the registration of patients through the computer random generation method.

### Study variables and measurement

The dependent variable was food security status, which was assessed cross-sectionally at baseline using a nine-item Household Food Insecurity Access Scale (HFIAS) developed and refined by the USAID Food and Nutrition Technical Assistance (FANTA) project (42, 43) and reported at the individual level. Considering the observation independence assumption, care was taken to not include more than one individual from the same household. The HFIAS is a validated instrument and has been shown to distinguish food insecure households from food secure households across different cultural contexts, considering the three dimensions of food security, such as (1) anxiety and uncertainty about household food supply, (2) insufficient quality (including variety and preferences of types of food), and (3) insufficient food intake and its physical consequences. The results were dichotomized into food insecurity and food security (16, 19, 44). Sociodemographic characteristics (age, gender, income, educational status, occupational status, religion, marital status, residence, number of children, and psychosocial supports) and clinical factors (duration of ART treatment, WHO clinical stage, WHO treatment stage of HIV, opportunistic diseases, therapeutic food support, and follow-up interval) were treated as independent variables in this study.

### Data collection tools and methods

A structured interviewer-administered questionnaire was developed to collect sociodemographic and HIV patient follow-up data. The questionnaire consisted of six parts, namely sociodemographic characteristics, psychosocial supports, clinical predictors and effects information, therapeutic food-related information, and household food security status. Food security data were collected, using a structured interviewer-administered questionnaire. Patient records were extracted to collect data on some variables, such as type of malignancy, IOs, anemia, and WHO staging. The questionnaire was pretested on 5% of the sample at Chancho Hospital for feasibility, consistency, and completeness in the population with similar characteristics. The necessary modification was made based on the result of the pretest before actual data collection. The content validation of the questionnaire with local experts was performed before adapting the FANTA food insecurity access scale. Experienced and qualified nurses, health officer data collectors, and supervisors were recruited and trained from those hospitals and health centers. Two days of training were given to data collectors and supervisors on the objectives of the study, methods of data collection, including the use of computer-assisted personal interviews (CAPIs) using KoboToolbox, and how to maintain the confidentiality of information. The CAPIs KoboToolbox digital data collection platform was used to collect data using data collectors on a digital platform. The measurement instruments were calibrated after every measurement. The collected data were checked for completeness and consistency.

### Data management and analysis

The data collected using the KoboToolbox digital data collection platform were exported to STATA 17 for cleaning and analysis, including modeling. Descriptive analysis was used to characterize the study variables. A log-binomial regression model was fitted to identify factors associated with food insecurity. All predictors associated with the outcome variable in bivariable analysis with a *p*-value of 0.20 or less were included in the log-binomial regression model of multivariable analysis. The crude and APRs, considered relative risk, together with their corresponding 95% confidence intervals, were computed. Multi-collinearity of explanatory variables was checked using the variance inflation factor, and the fitness of the model was checked. A *p*-value of <0.05 and corresponding 95% CI were considered to declare a result as statistically significant.

### Ethical consideration

The study protocol was reviewed and approved by the Institutional Review Board of the College of Health Sciences, Addis Ababa University, with a protocol number of 104/19/SPH. Permission for data collection was obtained from respective health facilities before data collection, and focal persons at ART clinics were informed. Study participants were informed about the purpose of the study and verbal informed consent was obtained from each study participant. The confidentiality of collected information was maintained by locking it in a file cabinet, accessible only by principal investigators. Participation in this study was voluntary, and participants had full right not to participate or withdraw from the study. The soft copy of data entered into a computer was stored in encrypted files on password-protected computers.

## Results

### Sociodemographic characteristics

A total of 865 adults who were HIV-infected and receiving antiretroviral therapy were enrolled, of those, 861 were willing and able to participate in this study, resulting in an overall response rate of 99.5%. The majority, 327(37.98%) of the participants were within the age group 35–44 years, and the mean age of the enrolled participants was 38.66 (±9.86SD) years. In terms of gender, 529 (61.44%) of them were women, and 781(90.71%) were Orthodox Christians. The majority of 615 (71.43%) participants were from urban areas. Six hundred forty-two (74.82%) were Oromo in ethnicity, and 522 (60.63%) were married. Six hundred ninety-two (80.37%) reported that they had children, of whom 507(73.27%) had less than 4 children, with a median of 3.0 (IQR: 2, 4; Table 1).

**Table 1:** Sociodemographic characteristics of HIV-infected adults receiving antiretroviral therapy at health facilities in North Shewa Zone, Ethiopia, 2023 (*n* = 861).

Variables	Frequency	Percent
Age of respondents		
<35 years	296	34.38
35–44 years	327	37.98
≥45 years	238	27.64
Mean(±SD) 38.66(±9.86)		
Gender of participants		
Male	332	38.56
Female	529	61.44
Religion of participants		
Orthodox	781	90.71
Protestant	55	6.39
Others*	25	2.90
Residence		
Rural	246	28.57
Urban	615	71.43
Marital Status		
Married	522	60.63
Single	96	11.15
Divorced	111	12.89
Widowed	132	15.33
Ethnicity of participants		
Oromo	645	74.91
Amhara	213	24.74
Gurage	3	0.35
Having children		
No	169	19.63
Yes	692	80.37
Number of children (*n* = 692)		
≤3 children	507	73.27
4–9 children	185	26.73
Median number of children	Median	IQR
Median (IQR)	3	(2, 4)

*Catholic, Muslim and Wakefata.

### Socio-economic characteristics

The distribution of occupational status is almost consistent across each category. Accordingly, 198 (23.00%) and 193 (22.42%) HIV-infected adults were merchants and housewives, respectively. Three hundred eighty-one (44.25%) of HIV-infected adults had no formal education. Two hundred ninety-two (52.52%) of HIV-infected adults reported earning a monthly income of 2,500 and above Ethiopian birr, with a median monthly income of 2500ETB (IQR: 1200, 4,730; Table 2).

**Table 2:** Socio-economic characteristics of HIV-infected adults receiving antiretroviral therapy at health facilities in North Shewa Zone, Ethiopia, 2023 (*n* = 861).

Variables	Frequency	Percent
Occupational Status		
Farmer	173	20.09
Housewife	193	22.42
Daily laborer	148	17.19
Employed	149	17.31
Merchant/others	198	23.00
Educational status		
No formal education	381	44.25
Primary school	240	27.87
Secondary and above	240	27.87
Monthly income (*n* = 556)		
<2,500 ETB	264	47.48
≥2,500 ETB	292	52.52
Median monthly income (ETB)	Median	IQR
Median (IQR)	2,500	(1,200, 4,730)

### Psychosocial support for HIV-infected adults

Of the total, most patients reported having some source of social support for informal caregiving. Accordingly, 417 (48.43%) of the HIV-infected adults reported that they received informal care from different caregivers. Of those, 273(65.47%) received economic support, followed by psychological support (86, 20.62%). Hundred fifty-nine (38.13%) received care from their husbands, followed by wives (86, 20.62%). Four hundred forty (51.10%) of the HIV-infected adults had disclosed their HIV status. Husbands (148, 33.64%), wives (96, 21.82%), and children (92, 20.91%) were the common categories of people to whom studied HIV-infected adults disclosed their HIV serostatus (Table 3).

**Table 3:** Psychosocial support for HIV-infected adults receiving antiretroviral therapy at health facilities in North Shewa Zone, Ethiopia, 2023.

Variables	Frequency	Percent
Presence caregiver (*n* = 861)		
No	444	51.57
Yes	417	48.43
Type of care received (*n* = 417)		
Psychological support	86	20.62
Economic support	273	65.47
Physical support	24	5.76
Social support	34	8.15
Type of caregiver (*n* = 417)		
Mother/ father	56	13.43
Wife	86	20.62
Husband	159	38.13
Children	81	19.42
Others	35	8.39
Disclose their Sero-status (*n* = 861)		
No	421	48.90
Yes	440	51.10
To whom you disclose (*n* = 421)		
Mother/father	46	10.45
Wife	96	21.82
Husband	148	33.64
Children	92	20.91
Community supporter	27	6.14
Others	31	7.05

### Clinical factors of HIV-infected adult

Only 43 (4.99%) reported they received therapeutic feeding in the courses of their treatment follow-up; of those, 37(86.05%), 3(6.98%), and 3(6.98%) received plumpy nut, food prepared for treatment, and high protein diet (eggs), respectively. They received therapeutic feeding treatment for 1 to 6 months. Hundred seventy-three (20.9%) of the patients developed eating problems; of those, 134 (77.46%) reported that they developed a loss of appetite. Only four (0.46%) of HIV-infected adults in the total sample reported malignancies, with one case of Kaposi’s sarcoma, two cases of cervical cancer, and one case of unidentified malignancy. One hundred seventy-three (20.09%) of the patients developed opportunistic infections (OIs) during their care follow-up, with the most common OIs being diarrheal disease (68, 39.31%), followed by tuberculosis (43, 24.86%). A significant proportion of studied participants reported that they developed anemia (212, 24.62%).

Six hundred ninety-six (80.84%) had been receiving ART and related care for more than 4 years, and the average amount of time that the studied HIV-infected adults received treatment was 9.2 years (±4.6SD). More than three-fourths, 672 (78.05%) of studied adults reported that the follow-up interval time was 3 and above months. The majority, 624(72.47%) and 764(88.73%) of the HIV-infected adults were at WHO clinical stage one and WHO treatment one, respectively (Table 4).

**Table 4:** Clinical factors of HIV-infected adults receiving antiretroviral therapy at health facilities in North Shewa Zone, Ethiopia, 2023.

Variables	Frequency	Percent
Therapeutic food (*n* = 861)		
No	818	95.01
Yes	43	4.99
Presence of eating problems (*n* = 861)		
No	688	79.91
Yes	173	20.09
Causes of Eating problem		
Loss of appetite	134	77.46
Oral candidiasis	33	19.08
Esophageal candidiasis	6	3.47
Opportunistic malignancy		
No	857	99.54
Yes	4	0.46
Presence of OIs (*n* = 861)		
No	688	79.91
Yes	173	20.09
Type of OI disease		
Tuberculosis	43	24.86
Pneumonia	31	17.92
Diarrheal disease	68	39.31
Dyspepsia	10	5.78
Others	21	12.14
Presence of anemia		
No	649	75.38
Yes	212	24.62
Duration of HIV infection		
<=5 years	192	22.30
>5 years	669	77.70
Average duration of HIV infection in years	Mean	SD
Average (SD)	9.76	4.65
WHO clinical stage		
Stage one	624	72.47
Stage two	167	19.40
Stages three and four	70	8.13
WHO treatment stage		
Stage one	764	88.73
Stage two	84	9.76
Stages three and four	13	1.51
Duration of ART		
≤4 years	165	19.16
>4 years	696	80.84
Average duration of ART in years	Mean	SD
Average (SD)	9.20	4.56
Follow up interval		
Less than 3 months	189	21.95
3 and above months	672	78.05

### The magnitude of food insecurity among PLHIV

**Figure 1:**
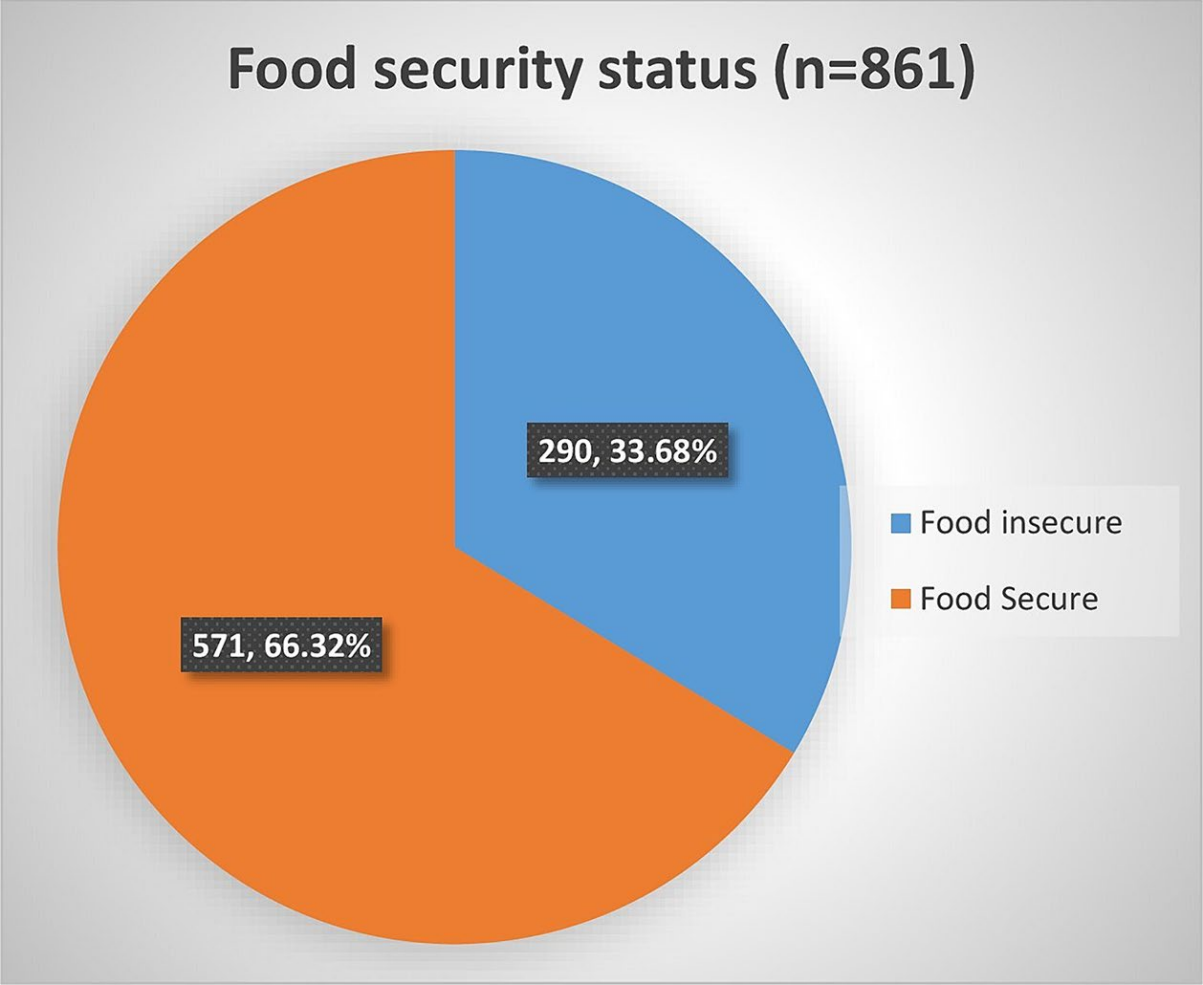
Household food security status of HIV-infected adult receiving antiretroviral therapy at health facilities in North Shewa Zone, Ethiopia, 2023.

The food security status of PLHIV receiving ART was assessed using food insecurity access score and prevalence indicators in this particular study. We used food insecurity indicators, specifically the Household Food Insecurity Access Scale (HFIAS) score and its prevalence indicator, to assess and report the status and severity of food insecurity. This involved utilizing nine items of HFIAS, along with questions regarding the frequency of occurrence. The prevalence indicators enabled us to assess and report the severity of food insecurity occurrence, categorizing households into four levels: food secure, mildly, moderately, and severely food insecure. Accordingly, 290 [33.68%; 95% CI: (30.60, 36.91)] were food insecure among adult HIV-infected patients receiving ART (Figure 1). Among those who were food insecure, 152 (52.41%, CI: 46.64, 58.13) of food insecure HIV-infected adults were found to have a severe form of food insecurity, followed by a moderate form of food insecurity, 110 (37.93%, CI: 32.50, 43.68; Table 5).

**Table 5:** Level of household food insecurity among HIV-infected adults receiving antiretroviral therapy at health facilities in North Shewa Zone, Ethiopia, 2023.

Variables	Frequency	Percent with CI
Severity of food insecurity (*n* = 290)		
Mild food insecure	28	9.66(6.74, 13.64)
Moderately food insecure	110	37.93 (32.50, 43.68)
Severely insecure	152	52.41(46.64, 58.13)

### Factors associated with food insecurity among PLHIV

We used log-binomial regression to identify the association between food insecurity and independent variables, a robust method of analysis for cross-sectional studies with binary outcomes, aiming to minimize the overestimation of the prevalence ratio. The prevalence ratio is also more interpretable and easier to communicate to non-specialists than the odds ratio.

Accordingly, in bivariable analysis, 17 variables—gender, age, marital status, occupational status, presence of children, number of children, monthly income, residence, educational status, type of caregivers, presence of OIs, types of OIs, duration HIV infection, duration of ART follow-up, ART follow-up interval, WHO clinical stage, and WHO treatment stage—showed association with a *p*-value of ≤0.2 and were selected as candidates for multivariable analysis. Five of the 17 variables, such as duration of HIV infection, presence of children, ART follow-up interval, type of caregivers, and types of OIs that showed collinearity with other related variables, were reduced after collinearity check using collinearity diagnostics such as correlation matrix and variance inflation factor.

Thus, the multivariable log-binomial regression analysis was performed, taking all variables into account simultaneously, and seven of the most contributing factors were significantly and independently associated with food security status at a 5% level of significance.

The gender of HIV-infected adults was found to have a significant and independent predictor of food insecurity, of which the proportion of food insecurity was 1.9 times higher among female subjects than male subjects [adjusted prevalence ratio (APR) = 1.87, 95% CI: 1.03, 3.39]. Food insecurity was higher among younger HIV-infected adults than older adults; the proportion of food insecurity was 2.3 times higher among those belonging to the age group less than 35 years than those belonging to the age group greater than 45 years (APR = 2.27, 95% CI: 1.12, 4.60). The educational status of the HIV-infected adults was found to have a strong significant association with food insecurity, of which the proportion of food insecurity was 11 and 6 times higher among HIV-infected adults who had no formal education and attended primary school, respectively, than those who attended secondary and above education (APR = 10.79, 95% CI: 14.74, 24.58) and (APR = 5.99, 95% CI: 2.65, 13.54).

Concerning occupational status, the proportion of food insecurity was 6.9 times higher among daily laborer patients than farmer patients when the effect of other variables was kept constant (APR = 6.90, 95% CI: 2.28, 20.85). The analysis found that lower monthly income leads to a higher proportion of food insecurity, among HIV-infected adults, of which the proportion of food insecurity was 1.9 times higher among HIV-infected adults who had monthly income less than 2,500 Ethiopian Birr than those who had monthly income greater than 2,500 Ethiopian Birr (APR = 1.89, 95% CI: 1.11, 3.22).

Among clinical factors, the WHO clinical stage and duration of antiretroviral treatment were strongly associated with a higher proportion of food insecurity among HIV-infected adults. The more advanced the WHO clinical stage, the higher proportion of food insecurity among adults was noted. Specifically, the proportion of food insecurity was 2.3 times higher among adults at WHO clinical stage two than the WHO clinical stage one (APR = 2.34, 95% CI: 1.08, 5.10). The proportion of food insecurity was 2.3 times higher among those receiving ART for less than 4-year duration (APR = 2.28, 95% CI: 1.09, 4.74). However, socio-economic support and food support/therapeutic food support were not significantly associated with the proportion of food insecurity in the final log-binomial multivariable regression (Table 6).

**Table 6:** Factors associated with food security status among HIV-infected adults receiving antiretroviral therapy at health facilities in North Shewa Zone, Ethiopia, 2023.

Variables	Food security status (No)		CPR with 95% CI	APR with 95% CI	*p*-value
	Food insecure	Food secure			
Gender					
Male	89	243	1.0	1.0	
Female	201	328	1.67 (1.24, 2.26)**	1.87(1.03, 3.39)**	0.039
Age category					
<35 years	120	176	2.02(1.39, 2.94)**	2.27(1.12, 4.60)**	0.023
35–44 years	110	217	1.50(1.04, 2.18)**	1.38(0.74, 2.60)	0.314
≥45 years	60	178	1.0	1.0	
Educational status					
No formal education	172	209	4.99(3.29, 7.55)**	10.79(4.74, 24.58)**	<0.001
Primary school	84	156	3.26(2.08, 5.11)**	5.99(2.65, 13.54)**	<0.001
Secondary and above	34	206	1.0	1.0	
Occupational status					
Farmer	32	141	1.0	1.0	
Housewife	64	129	2.19(1.34, 3.56)**	0.95(0.32, 2.80)	0.921
Daily laborer	106	141	11.12(6.58, 18.79)**	6.90(2.28, 20.85)**	0.001
Employed	30	119	1.11(0.64, 1.93)	1.22(0.40, 3.74)	0.728
Merchant/others	58	140	1.83(1.12, 2.98)**	1.2(0.37, 2.83)	0.965
Monthly income(ETB)					
Less than 2,500	144	120	3.74(2.61, 5.36)**	1.89(1.11, 3.22)**	0.020
2,500 and above	71	221	1.0	1.0	
WHO clinical stage					
Stage one	223	401	1.0	1.0	
Stage two	50	117	0.77(0.53, 1.11)*	2.34(1.08, 5.10)**	**0.032**
Stages three and four	17	53	0.58(0.33, 1.02)*	2.25(0.69, 7.33)	0.179
Duration of ART					
≤4 years	81	84	2.25(1.59, 3.17)**	2.28(1.09, 4.74)**	0.028
>4 years	209	487	1.0	1.0	

^∗^Mean factors that show association with FAVs at *p*-value less than 0.25 (rule of thumb). ^∗∗^Mean factors that show statistically significant association with FAVs at *p*-value less than 0.05. Bold values indicates those factors that showed statistically significant association with food insecurity.

## Discussion

This study aimed to determine the magnitude of food insecurity, its severity, and its associated factors among HIV-infected adults receiving ART. We found that one-third [33.7%: (30.60, 36.91)] of the HIV-infected adults living in the household have food insecurity. Gender, early age, occupational status, educational status, monthly income, WHO clinical stage, and duration of ART were significantly and independently associated with food insecurity.

The magnitude of food insecurity in this study is in line with the study conducted in the West Shoa Zone (35.2%) (31) and in Kenya (33.5%) (45). However, the finding is lower than the finding of studies in Ethiopia, particularly studies conducted in Benishangul Gumuz (76%) (46), hospitals in western Ethiopia (68.8%) (47), Debre Markos Town (84.52%) (29), Hosanna (67.53%) (28), (68.48%) (27), Tigray (40.43%) (48), studies in Jimma (63.01%) (30), (85.92%) (24), Kembata Tembaro (57.3%) (49), and studies in Butajira (78.11%) (25), (79.02%) (26). The difference may be attributed to several factors, including the time gap between previous studies and the current one, as well as the larger sample size included compared to previous studies. As a multi-center study, the inclusion of multiple study sites ensured the representativeness of the area, which could have contributed to the variation in the findings. The magnitude of food insecurity in this study was also lower than in some previous studies in African countries. For instance, among PLHIV, rates were reported as 60% in South Africa (50), 57% in the Democratic Republic of Congo (19), 84.6% in Dakar (20), and 74.6% (51) and 38% (52) in two studies in rural Uganda. The long duration since the previous studies were conducted, the socio-economic characteristics of the population, and the cultural context of the study areas are considered to be possible explanations for the difference in the level of food insecurity.

The magnitude of food insecurity in the current study is higher than the findings of the studies conducted in Dembia Gondar (18.36%) (53) and Arba Minch (19.54%) (36). The possible explanation may be the smaller sample in the previous studies and the timing of the studies.

We find that 52.4% (46.6, 58.1) of HIV-infected adults from households living with food insecurity were found to have a severe form of food insecurity, which is higher than the findings of studies in Western Ethiopia (16.35%) (47), Kembata Tembaro Zone (17.4%) (49), and two studies in Butajira (42.0%) (25) and (41.7%) (26). This highlights the need for critical care and support attention during ART follow-up, as only 5% of the participants reported that they received food support, including nutritional counseling.

We found a strong and significant association between the gender of HIV-infected adults and food insecurity, in which the proportion of food insecurity was two times higher among female subjects. This finding is not surprising and is in line with those recent studies on the topic. For instance, a systematic review published early in this study by the principal author of Onyenakie et al. (54), a study conducted in Arba Minch (36), and another study conducted in the Dominican Republic (35) (have reported similar findings). However, it requires special attention in a country like Ethiopia, where women often face greater social and economic disadvantages, potentially aggravating their food insecurity situations.

Our study found that age and magnitude of food insecurity were significantly associated, with the proportion of food insecurity being higher in the younger age group compared to the older age group. Surprisingly, we found mixed evidence regarding age as a contributing factor to food insecurity among HIV-infected adults. For instance, the younger age of patients was associated with an increased proportion of food insecurity in the study conducted in Brazil and other high-resource settings (18, 56) as well African countries (55), while an increased proportion of food insecurity was found among older adults in the United States (56), and some studies indicated a lack of significant association between patients’ age and food insecurity. This indicates the need for further study on the association between the age of HIV-infected adults and food insecurity.

This study found a significant and independent association between the monthly income of HIV-infected adults and food insecurity, which stipulated that the lower the monthly income, the higher the proportion of food insecurity among HIV-infected adults. This finding was fairly established and in line with findings of previous studies conducted in Western Ethiopia (47), Hosana Town (28), Kembata Tembaro Zone (49), Arba Minch (36), Butajira (26), and rural Zambian hospitals (57).

We found a strong and significant association between the proportion of food insecurity and the educational status of HIV-infected adults, in which the proportion of food insecurity was higher among HIV-infected adults who had no formal education and attended primary school. The finding is in line with other previous studies that have found no education and lower levels of education to be strongly associated with food insecurity in Western Ethiopia (47), Hosanna Town (28), Brazil (18), Jimma Zone (30), and Nigeria (22).

In the current study, the lack of permanent employment was found to have a significantly strong association with the proportion of food insecurity being higher among patients who were daily laborers. It may be the result of a lack of permanent employment, which can affect the earning capacity of HIV-infected adults and, consequently, increase the proportion of food insecurity. The finding is supported by the finding of the previous studies that unemployment was found to have a significant association with food insecurity in Hosanna Town (28), Nigeria (22), and Brazil (18).

Concerning clinical factors, we found a significant and independent association between the WHO clinical stage and the duration of antiretroviral treatment among HIV-infected adults. The proportion of food insecurity among patients with advanced WHO clinical stage and receiving ART for less than 4-year duration was found to be high. This may be due to worsening disease situations and deteriorated health status at the advanced clinical stage of HIV with delayed ART initiation. The significant association of food insecurity with advanced clinical stages is supported by previous studies conducted in different areas and settings, for example, in Ethiopia (27, 32, 36, 49, 58). In the same way, a significant association with the duration of ART is also supported by the findings of studies conducted in Africa, including Ethiopia (59) and Namibia (34), in which the shorter duration of ART and the high proportion of food insecurity among HIV-infected adults have been demonstrated. This will imply that longer ART duration, greater than 4 years, may be associated with lower food insecurity, possibly due to improved health status and coping capacity among HIV-infected adults receiving ART. However, it is not sufficient to establish a cause-and-effect relationship using a cross-sectional survey alone without evidence from a follow-up study.

However, we found a lack of significant association between food security and socio-economic status and food support in the final log-binomial multivariable regression, while the absence of support was found to be strongly associated with food insecurity in South Wollo (58), Kembata Tembaro and Hosanna Town (27, 49), and the Dominican Republic (35). The lack of a significant association between therapeutic feeding supports in the current study may be due to the fact that a very small proportion of HIV-infected adults received food by prescription.

## Limitations of the study

The current study assessed the food security status of HIV-infected adults by including all health facilities with established ART clinics with diverse service delivery levels and using a large sample size to determine the number of HIV-infected adults receiving ART. The KoboToolbox digital data collection platform allows the investigator to follow and approve the collected data daily, train data collectors and supervisors, and use a log-binomial model suitable for estimating prevalence ratios. This ensures the credibility of the evidence generated in this study. However, the current study has some situational and methodological limitations. The first is the use of a cross-sectional study design, in which data were collected at a point in time for both independent and dependent variables for this specific objective. This temporal relationship may limit the ability to identify causal and effect relations between food insecurity and independent variables. The second is the possibility of recall bias, particularly in food security status assessments, as they were asked about their experience over the last 30 days. Third, given the scope of the current study, the relationship between food insecurity and loss of follow-up among HIV-infected individuals was not explored. Further comparative research is required to analyze food insecurity differences between those lost to follow-up and those not lost and their impact on loss to follow-up. Fourth, there is a possibility of social desirability bias due to the sensitivity of the issues under study and the limited cultural tendency in our societies to discuss such sensitive issues frankly.

## Conclusion

The findings of this study indicate that the magnitude of food insecurity among HIV-infected adults receiving ART was high, with extremely severe forms of food insecurity. This is also indicated as its magnitude is still a critical public health problem in the society living in areas with surplus crop production. The gender of HIV-infected adults, being younger, occupational status, educational status, monthly income, WHO clinical stage, and duration of ART were significantly and independently associated with a high proportion of food insecurity among HIV-infected adults. The findings suggest the importance of education for HIV-infected people, attention to the early stage of ART, and advanced clinical stages of patients in the form of patient education and nutritional counseling for patients with advanced clinical stages of the disease. The nutritional intervention should be culture- and context-specific to address the gender dynamicity of food insecurity among HIV-infected adults. The findings also suggest the need for emphasis on the link between food insecurity and HIV in Ethiopia’s National Food and Nutrition Policy.

## Data availability statement

The raw data supporting the conclusions of this article will be made available by the authors, without undue reservation.

## Ethics statement

The study involving humans were approved by the Institutional Review Board of the College of Health Sciences, Addis Ababa University, with a protocol number of 104/19/SPH. The study were conducted in accordance with the local legislation and institutional requirements. Written informed consent to participate in this study was not required from the participants in accordance with the national legislation and the institutional requirements. The participants provided their verbal informed consent to participate in this study.

## Author contributions

DB: Conceptualization, Data curation, Formal analysis, Investigation, Methodology, Project administration, Software, Supervision, Visualization, Writing – original draft, Writing – review & editing. AA: Conceptualization, Data curation, Investigation, Methodology, Software, Supervision, Validation, Visualization, Writing – review & editing. AY: Conceptualization, Data curation, Investigation, Methodology, Software, Supervision, Validation, Visualization, Writing – review & editing.
